# Gender Diverse Children and Adolescents in Italy: A Qualitative Study on Specialized Centers’ Model of Care and Network

**DOI:** 10.3390/ijerph17249536

**Published:** 2020-12-19

**Authors:** Guido Giovanardi, Alexandro Fortunato, Marta Mirabella, Anna Maria Speranza, Vittorio Lingiardi

**Affiliations:** Department of Dynamic and Clinical Psychology, Sapienza University of Rome, 00185 Rome, Italy; alexandro.fortunato@uniroma1.it (A.F.); marta.mirabella@uniroma1.it (M.M.); annamaria.speranza@uniroma1.it (A.M.S.); vittorio.lingiardi@uniroma1.it (V.L.)

**Keywords:** gender incongruence, specialized centers, transgender youths, qualitative study

## Abstract

In recent years, Italy, similar to many other countries, has witnessed an increase in children and adolescents presenting gender incongruence. This trend has led to the development and implementation of specialized centers providing care and support for these youths and their families. The present study aimed at investigating the functioning of agencies specialized in working with transgender and gender non-conforming youths in the Italian territory. Professionals in these agencies were interviewed about their perspectives on their agency’s functioning, networks with other services, and work with trans* youths and their families. A semi-structured interview was developed and administered to professionals in specialized centers and associations dedicated to trans * youths, and deductive thematic analysis was applied to the transcripts. Eight professionals were interviewed: six working in specialized centers and two working in associations. The qualitative analyses of transcripts revealed four main themes, pertaining to service referrals, assessment protocols and intervention models, psychological support for youths and families, and agency shortcomings. The study explored the functioning of Italian agencies specialized in caring for transgender and gender non-conforming youths, from the perspective of professionals working in these agencies. While several positive aspects of the work emerged, the study highlighted a lack of uniformity across the Italian territory and the need for better networks between agencies and other medical professionals.

## 1. Introduction

In 2011, the World Professional Association for Transgender Health (WPATH) published the seventh edition of its Standards of Care for Transgender Health (SOC), underlining the importance of adopting a multidisciplinary approach in the care of children and adolescents with “gender incongruence” (In line with ICD-11 [1] and PDM-2 diagnoses [2], we use the term “incongruence” over “dysphoria”. We chose to use the term “trans*” in place of “transgender” or “transsexual” to use a broader term that encompasses several conditions related to gender diversity.) (GI) [1]. Their recommended approach consisted of psychological interventions for minors and their families and physical interventions for adolescents, provided by qualified mental and physical health professionals [3]. Subsequently, in 2015, the American Psychological Association (APA) published its Guidelines for Psychological Practice with Transgender and Gender Nonconforming People [4], with the aim of providing clinical guidance for health professionals working with transsexual, transgender, and gender non-conforming people (including youths). These guidelines were designed to engender safe and effective pathways to support transgender and gender non-conforming persons in developing comfort with their gendered selves, to maximize their overall health, psychological well-being, and self-fulfillment. The guidelines encapsulated primary care, gynecologic and urologic care, reproductive options, mental health services (e.g., assessment, counseling, psychotherapy), and hormonal and surgical treatments [5].

A recent review of the literature suggests that 0.17–1.3% of adolescents and young adults may identify as transgender [6]. Internationally, several specialized centers have been established for their care [7,8], and, over the last decade, these centers have seen a steep increase in referrals [9,10,11]. For instance, at the Gender Identity Development Service (GIDS) in London (UK), the number of referred children and adolescents grew from 51 to 1766 between 2009–2016 [12]; a similar trend was also observed in Amsterdam [7]. As many studies have reported, there is an evident bias in the sex ratio of referrals to these centers, showing a slight preference for assigned females at birth (AFAB) over assigned males at birth (AMAB) [9,10,11,12,13]. Additionally, some authors have reported new presentations of gender incongruence among recent referrals—especially AFABs—characterized by “rapid onset,” with feelings of gender dysphoria presenting rapidly around the time of pubertal changes [8,14,15]. However, the notion of rapid onset has been criticized by some authors, and requires further research [16].

Children and adolescents who are referred to specialized centers for gender incongruence usually present a range of mental health issues [8,17,18,19,20]. Generally, the difficulties reported in the literature are internalizing in nature (e.g., depression, anxiety, eating disorder) [6,18,19]; however, there is considerable variability in the results across studies [17]. Research has shown that trans youths are at higher risk of self-harm, suicidal ideation, and suicidal attempts [21,22,23,24,25,26,27,28,29], and that the well-being of these youths can be hindered by “internal” sources, such as body dissatisfaction or distress [30,31,32,33,34]. As Ristori and Steensma [17] noted, a significant proportion of these psychological problems may be understood as a response to the discrimination experienced by gender diverse youths, as conceptualized by the minority stress model [35]. Frequently, families and social environments are not prepared or knowledgeable about gender diversity, and this may give rise to stigma or discrimination against trans youths [36,37,38]. Moreover, some trans people have reported discrimination from healthcare professionals [39,40,41]

The population of gender diverse youths who are referred to gender clinics has significantly changed over recent years, and it is continuing to develop, manifesting new needs and requests. Many relevant topics to this population (e.g., the use of hormone blockers [42]) are the subject of animated debates within the scientific community over the best practices of care and support [8,43,44,45,46]. More research is required to gain insight into these subjects and thereby support these youths.

In Italy, specialized centers for trans * youths are networked within the Osservatorio Nazionale sull’Identità di Genere [National Observatory on Gender Identity] (ONIG), which, together with several scientific societies (e.g., the Italian Society of Andrology and Sexual Medicine, the Italian Society of Endocrinology, the Italian Society of Pediatric Endocrinology and Diabetes), established a consortium of professionals engaged in the clinical, endocrine, legal, and social management of trans * youths. In 2014, these societies promoted specific guidelines for centers and professionals to provide the best care and support to gender variant and transgender individuals [47,48]. These guidelines are now followed by the specialized centers connected with the Italian National Health Service (Sistema Sanitario Nazionale; SSN), which usually adopt a staged approach consisting of: (1) a psychodiagnostic procedure to assess gender diversity and general psychological functioning; (2) suppression of puberty for eligible adolescents (which, to date, they have only applied to a few persons under 16 years old); and (3) cross-sex hormone therapy for adolescents aged 16 and older and gender reassignment surgery (GRS) for adults (aged 18 years and older), if requested.

In Italy, uniform application of these guidelines is impeded by some shortcomings related to the SSN, which was established in 1978. The SSN is based on the principles of universalism, comprehensive care, progressive tax funding, and resource efficiency. Its functions are decentralized, with management devolved to 21 regions and autonomous provinces. However, the funding for this service is not stable, and it faces chronic deficits. Furthermore, regional differentiation of the national healthcare model has had important consequences for regulatory arrangements [49]. Most notably, the distribution of assets and funds for regional healthcare is not uniform, and there are critical divides between northern and southern regions [50,51].

In line with this, the distribution of specialized centers for trans * youths across the Italian territory is not even: such centers are only present on the mainland, leaving large portions of the country (e.g., Sicily, one of Italy’s largest regions) without cover; many major metropolises (e.g., Milan) are similarly lacking. In some areas, voluntary unions and LGBT groups have organized to fulfil some of the work performed by clinical centers (e.g., reception of referrals, psychological support, development of peer groups). Moreover, within the Italian territory, regional connections are poor (e.g., between the islands and the mainland, or between the eastern and western regions), and travel between regions can take significant time. Thus, for many families, it is not feasible or affordable to travel to specialized centers, especially considering that, consistent with the standards of care [3,4,5], trans * youths require close and continuous attention by professionals in these centers.

Recent research has shown that, in Italy, the population of trans * youths [52,53] is increasing. Several studies highlighted the risk for this population to suffer traumas and mental health problems due to minority stress and discriminations [54,55,56,57,58,59,60]. This is adding pressure to existing centers and highlighting the need for a more extensive network of care and support. Within this context, the work of clinicians in the specialized centers has remained understudied. While previous Italian qualitative studies have investigated the experiences of trans * individuals [61,62], their families [63], and clinicians working outside of specialized services [64], to the best of our knowledge, the present study was the first to investigate the perspectives of clinicians and volunteers within specialized centers and associations for trans * youths, with respect to agency functioning and shortcomings.

Our aim was thus to gain a deeper understanding of the model of care provided to trans * youths in Italy within this context of rapid change and territorial complexity, investigating in particular:

(a) the intervention model and functioning of the specialized centers and associations involved in the care of trans * youth;
(b) the networks of each service with other services and the relationship with the broader Italian social context;
(c) the psychological work done with youths and families in the Italian specialized services.

## 2. Materials and Methods

### 2.1. Measures

The data for this study derived from eight semi-structured interviews with experienced professionals (psychologists and social workers) working in specialized centers and in associations for the care of trans * youths. The semi-structured interview was developed by first three authors and entailed questions about: (1) the general characteristics of the referrals observed at the service where they work (number per year, recent trends, prevalence regarding age and gender, etc.); (2) from whom, more frequently, were users referred to the service; (3) if the service took part in national or international research protocols; (4) what is the protocol of interventions/treatments and if the service adheres to specific guidelines; (5) description of the psychological work offered by the service; (6) descriptions of the involvement and reactions of parents and families; (7) the narration of a clinical case.

All subjects gave their informed consent for inclusion before they participated in the study. The study was conducted in accordance with the Declaration of Helsinki, and the protocol was approved by the Ethics Committee of Department of Dynamic and Clinical Psychology, Sapienza University of Rome, Italy (Project accepted on 1 February 2017).

### 2.2. Procedure

All interviews were audiotaped and transcribed verbatim, with identifying information removed. The interview transcripts were analyzed using deductive thematic analysis [49,50], to facilitate an interpretation of themes within the complex cultural context. Each transcript was imported into the software NVivo (release 1.3.1, QSR International, United States, Canada and Latin America), read and reread for familiarization, by G.G., A.F. and M.M.. Transcripts were coded using a deductive approach [65,66] and analyzed using thematic analysis. Each transcript was coded separately by three researchers (G.G., A.F. and M.M.) and discussed until a consensus on each code was reached. Unclear or opposing understandings of the interviews, were validated further by discussions with researchers A.M.S. and V.L.. After initial coding was established an iterative process began of reviewing and revising the codes as the overarching codes began to develop. This led to the development and refinement of four structural themes, ”source of referrals”, ”assessment protocols and intervention models”, ”psychological support (for youths and families)”, and ”agency shortcomings”, which each contained multiple sub-themes.

### 2.3. Participants

All Italian services (four in the north, four in the center, and two in the south) were contacted and invited to take part in the research, but only eight services that treat youths agreed to participate and provide information on their functioning. These eight services included six centers affiliated with the SSN (one in the north, three in the center, and two in the south) and two private associations (one in the north and one in the center). All interviews took place between March 2017 and December 2019. The professionals involved were psychologists and social workers, all with at least 2 years of experience working in their agencies.

## 3. Results

Four themes emerged from the thematic analysis of the interview transcripts: (1) source of referrals (2) assessment protocols and intervention models, (3) psychological support (for youths and families), and (4) agency shortcomings. Each theme included a number of subthemes (see Table 1).

### 3.1. Source of Referrals

The first emergent theme pertained to the source of referrals to the specialized centers and associations. Overall, clinicians described a varied scenario, with significant differences between centers. Nonetheless, the main sources of referrals were described as Internet forums and word-of-mouth:

“*Internet is where they discover us. Psychologists and pediatricians do not know us. Our referrals usually find us on online forums, and they come to us complaining about this scarce knowledge among primary care clinicians.*”

(SC, S; see Table 2 for the code legend)

Some specialized centers—especially those in the center of Italy—reflected a greater variety of sources, including direct referrals from pediatricians, psychologists, psychotherapists, and other SSN professionals:

“*In our region we are very well known. We receive referrals from everywhere: hospitals, schools, families… we are well grounded in our territory*”.

(SC, C)

These discrepancies highlight a lack of uniformity across the Italian territory regarding knowledge about the existence of specialized centers and, more generally, gender incongruence. In regions that are lacking in knowledge, there may be a greater risk of stigma and discrimination towards trans * youths by medical staff [39]. Indeed, many respondents reported complaints from their patients about previous encounters with uninformed general practitioners (GPs):

“*Experiences with GPs can be traumatic. One patient reported that his GP told him that “certain things” happen only in Casablanca.*”

(A, N)

Furthermore, respondents typically reported that their patients faced economic and logistical challenges in reaching the specialized centers, especially in the south, where fewer services were available:

“*For many families, organizing regular visits to a specialized clinic in a nearby city (up to 250 km away) is too difficult and expensive. Thus, we try to arrange visits to specialists in their surrounding areas, who have some knowledge of the topic. But these specialists are hard to find!*”

(A, C)

Regarding patients, clinicians confirmed the international trend [9] of growth in recent years. In particular, they reported an increase in AFABs presenting around puberty. The request of these patients was very clear: to start hormone therapy soon, to prevent pubertal changes:

“*We have long waiting lists, but with our resources we cannot see more than two new young persons per week. In recent years, we have seen a big change in referrals: many more AFABs, coming to us around puberty with a strong desire to prevent it.*”

(SC, C)

### 3.2. Assessment Protocol and Intervention Model

As already mentioned, the scientific community has not yet achieved consensus over the best clinical practice with trans * youths. In the literature, different intervention models are proposed [8,17,67,68], demonstrating great variety. The most widely endorsed of these models was developed in the mid-1990s by Dutch clinicians [69], and is often referred to as “watchful waiting.” This approach consists of interventions focused on increasing knowledge about gender incongruence and supporting the child and family as the child’s gender identity naturally unfolds. Other approaches vary from more conservative approaches [70] focused on lessening the dysphoria linked to gender incongruence and discouraging social transitions or name changes prior to puberty, to more liberal approaches that affirm the child’s gender role change from an early age [71].

Our respondents seemed to endorse the Dutch model: they agreed on a gender affirmative intervention, based on the principles of “do no harm” and “watch and wait” until the onset of puberty; at the same time, they aimed at supporting youths and families in understanding the subjective meaning attributed to gender identity and helping them face stigma and discrimination [72,73,74]. With regard to assessment, some centers completely adhered to the staged approach promoted in the WPATH and ONIG guidelines. This was true of all SSN centers, for instance, which were well-connected with endocrinologists and other relevant medical units. For the initial patient evaluation, clinicians used a battery of tests that were also frequently employed in other international centers:

“*Let’s say that we embrace the Dutch protocol. We start with a diagnosis using several psychodiagnostic tests, and then, when incongruence is present, we proceed with affirmative therapy, including the usage of hormone blockers—to date only a few cases, with the approval of our ethics committee—and then when they turn 16 they can start with cross-sex hormones, and then surgeries.*”

(SC, C)

However, in contrast to the practices of some international centers (e.g., GIDS in London), many clinicians declared that their service tended to reject care to youths presenting severe psychiatric conditions, whom they referred elsewhere:

“*When the kid arrives, we perform psychodiagnostic evaluations with several personality tests. When needed, we organize a deeper evaluation of psychiatric complications with a child psychiatrist. If there are no severe psychiatric problems, we produce the diagnosis and refer them to the endocrinology unit to begin hormonal treatment.*”

(SC, C)

It should be underlined that only a few clinicians declared that their center adhered to a specific and standardized protocol for assessment and intervention. Reported barriers usually referred to a lack of strong connections to medical units (e.g., endocrinologists, psychiatrists) and an absence of multidisciplinary staff. Thus, the inability to detect the presence of a severe psychopathological condition at intake was considered a critical shortcoming:

“*A multi-disciplinary evaluation would be great, but we have various problems in finding the right specialists: in our service, we only have psychologists and volunteers; no other medical units. We need to find them in external hospitals, and these connections are never easy.*”

(SC, N)

Moreover, many clinicians affirmed that they tended to privilege clinical interviews over the administration of psychodiagnostic tests:

“*Let’s say that we prefer to use only clinical interviews to assess the gender identity of the young person. Tests are useless: they are old and obsolete, and the kids already know the answers! Tests tend to pathologize, whereas interviews put the person at ease and enable us to learn more about her/him.*”

(SC, S)

### 3.3. Psychological Support (for Youths and Families)

The WPATH SOC7 [3] suggests that, in specialized multidisciplinary clinics, trans * youths should have ongoing contact with mental health professionals for the duration of their transition path. Regarding psychological support, the guidelines state that “psychotherapy should focus on reducing a child’s or adolescent’s distress related to the gender dysphoria and on ameliorating any other psychosocial difficulties” [3] (p. 175). Our respondents confirmed that, in their agencies, psychological support was fundamental. Such support was directed at three main targets: (1) the youths’ mental health and psychopathological correlates, (2) families, and (3) schools and peer groups. Regarding psychopathological correlates, clinicians confirmed the results reported in the literature, highlighting predominantly internalizing problems in patients; such problems were reported to improve during transition. Of interest, in some cases, therapeutic work revealed that patients’ psychopathological aspects served to control the body and prevent pubertal changes, as in the following example of a patient with an eating disorder:

“*One trans girl arrived at age 13. She suffered from severe anorexia nervosa. We networked with a clinic for eating disorders, and after she had been in recovery for some months, she came to us for hormones and psychological support. I think that psychological support, monitoring and work with hormone blockers were key to overcoming her eating disorder. She understood that her anorexia was a means of blocking her bodily change, “to not grow up as a male,” in her words. With the help also of family therapy, her psychopathological symptoms receded. Now she’s 17 and she lives a happy life.*”

(SC, C)

Many respondents emphasized the importance of working with families, who typically presented severe anxiety regarding the gender incongruence of the child or adolescent. As the literature reports, trans * youths often lack support from parents and peers [38,39,40,41,42,43,44,45,46,47,48,49,50,51,52,53,54,55,56,57,58,59,60,61,62,63,64,65,66,67,68,69,70,71,72,73,74,75]. In fact, several studies have shown correlations between family rejection and negative health outcomes, including depression, suicidality, and drug and alcohol use [76,77]. Respondents reported that families tended to be anxiously preoccupied with the potential social response to their child’s transition, including any problems the child might encounter at school, at university, or on the job market:

“*Recurrent fears of parents are those regarding work placement and the future of their children, in general. At first they are usually very scared, worried about prejudices in the Italian society. Some have high levels of transphobia, themselves, thinking of their child as very sick, or doomed to have a sad and dangerous life. Step by step, mostly thanks to parents’ groups, they become more accepting and open.*”

(SC, C)

Many parents also worried that their children might not find a loving partner or a stable relationship. In some cases, the agencies’ psychological work with parents focused on helping them to elaborate the loss of their child’s natal gender (and usually the anticipation of becoming a grandparent). Consistent with the literature [64,65,66,67,68,69,70,71,72,73,74,75,76,77,78], mothers tended to appear less frightened than fathers, and both parents were reported to have concerns related to a lack of knowledge on how to act:

“*They [mother and father] usually disagree on everything. Mothers tend to blame themselves, judging themselves as having been too cold or too suffocating… fathers might be very detached at first, or too scared. More than anything, they are both uncomfortable and unaware. They need information on their child’s condition, which for most of them is really an enigma.*”

(A, N)

Respondents agreed that, over recent years, families had begun to show greater acceptance of their child’s perceived gender. Usually, despite an initial refusal, families became very supportive of the child’s developing path after only a few months of family psychotherapy or parents’ groups. This is very important, since, in this population, positive parental attitudes and positive relationships with the family are associated with higher self-esteem and fewer suicide attempts [38,79,80]:

“*I have to say that parents who do not accept their child’s gender identity are becoming very rare. In many cases, it just takes a bit of time for the family to face their fears and learn to accept and support their children completely. Most of their fears, more than their own prejudice, are related to external society, which they think will never fully accept transgender people.*”

(A, C)

Finally, some respondents described their agency’s efforts to establish a network with schools and peer groups. In some cases, the agencies had succeeded in doing so, generating good outcomes for their patients’/users’ self-esteem and social acceptance. In other cases, a lack of resources made school collaborations difficult to achieve.

“*Usually with schools I immediately get in touch with the class or school coordinator and give her/him the necessary information. Sometimes I produce a certificate with the diagnosis, in which I suggest the use of correct names and pronouns at school, or recommend that the young person be allowed to use their preferred toilet. The responses from schools vary a lot, from those who agree on the name change (also on registers) to those who disagree completely, who are usually afraid of the reactions of the other children or parents.*”

(SC, N)

“*We would love to interact with schools. It is really necessary for some kids. Unfortunately, we rarely have the opportunity to organize groups or have a continuous relationship with school professionals.*”

(SC, S)

### 3.4. Agency Shortcomings

The final theme comprises all of the shortcomings highlighted by respondents. In line with our expectations (as articulated the Introduction), some discrepancies emerged between agencies in different Italian regions. In many cases, centers and associations were cut off from other medical services. Where this occurred, the agencies were significantly limited in their ability to network with other units, and they were therefore unable to provide a broad range of services to patients. In particular, respondents from associations complained of a lack of local resources. While they were able to provide counselling, peer support, and information about transition paths, they typically lacked any connection to a specialized center or hospital:

“*We have neither hospitals nor specialized centers around here (the nearest is 300 km away!), so we organize peer groups and psychological counselling. However, when our users begin hormone therapy far away, it is difficult to maintain a relationship with them and give them the support they need.*”

(A, C)

On their part, specialized centers often reported a lack of internal medical resources. For instance, these centers did not include a psychiatric unit, so they had to refer patients externally for psychiatric evaluation. There was a significant risk that these external evaluators would have little to no knowledge about gender incongruence:

“*It happens that we have to refer the family to psychiatrists we do not know, and sometimes they are very negative towards gender transitions, and may discourage or even scare the family about the consequences for their child. In other cases, external professionals are very open and affirmative, but it may still be difficult for the child to perform another “coming out” in front of another mental health professional.*”

(SC, C)

A lack of resources also hindered centers from collecting research data and participating in national or cross-national studies, which might have helped them to establish important connections with international centers:

“*Lacking internships, students, PhDs… it is very difficult to collect, organize, and send research data to research groups. We would like to participate in many projects, but we really cannot afford it!*”

(SC, C)

Finally, in line with the literature [8], clinicians reported that, in their work with adolescents—especially trans boys (AFABs)—patients tended to arrive at the service with a clear and insistent request for hormonal treatment as soon as possible. Most respondents agreed that, especially during the first stages of adolescence (ages 11–13 years), patients needed to explore their personal sense of gender identity in depth. Thus, psychological work usually consisted in buying time for the youths to gain a better comprehension of their condition and for their families to process the changes:

“*There are many Internet forums where they get all the information to compile the questionnaires and obtain the “good results” for a diagnosis. So they visit us with the idea that, as soon they arrive, they will start with hormones. But it takes time. It takes time even for the request for blockers to be approved by our ethics committee. It takes a bit of time to explore the meaning they give to their gender identity*”.

(SC, S)

“*They want everything, and they want it now. They do not want to live “the transition,” they just want to go from A to Z. They are scared by the stigma and discrimination they might suffer during transition. Italy is still not ready for non-binary people or people in transition! So our work is usually to slow down or “put on the brakes,” to help young persons and families not do things in a hurry, inappropriately, but to give them the tools to counteract transphobia and really support the child.*”

(A, N)

## 4. Discussion

The present study aimed at exploring Italian specialized centers and associations working with trans * youths, to investigate the functioning of their services, their models of intervention, and the shortcomings of their work with this population. A complex scenario emerged, suggesting that, despite the attempt to provide support in accordance with widely endorsed models and guidelines, there is not yet an Italian consensus on the best approach to working with gender incongruence. Considering the qualitative nature of our design research, we underline that our findings should be examined only as exploratory data.

First of all, a difference between specialized centers and associations emerged, with the latter ones providing only limited services (e.g., support groups, psychological help) and usually being more isolated and not well connected to medical units.

With regards to the referrals, in line with international trends [9,10,11,12], Italian’s population of trans* youths seem to be growing, particularly with respect to AFABs. Some respondents depicted referrals with traits of the so-called “rapid onset” [15] of gender incongruence, especially when describing AFABs, with pressing requests to start soon hormone therapies and an (apparent) lack of history of gender incongruence. However, this is a very complex phenomenon that needs further exploration. For instance, we cannot know if the lack of an earlier onset of gender incongruence was due to the fear of coming out in an unaccepting environment.

As noted in the Introduction, specialized centers may significantly differ on the basis of their connections to the SSN and/or other medical services. For instance, these agencies may benefit from different referral pathways. In the present study, all agencies reported a significant number of referrals via Internet forums and word-of-mouth, highlighting a general lack of knowledge in the broader Italian society regarding gender incongruence and services specialized in dealing with it, confirming what previous studies on Italian society found out (e.g., [56,57]). However, specialized centers connected with the SSN received a greater number of referrals from other medical professionals (e.g., pediatricians, general practitioners, psychologist, psychotherapists). This illustrates the lack of consistency in the care offered to trans * youths throughout the Italian territory: since the SSN is funded and organized on a regional basis, SSN networks vary significantly throughout the country. In the present study, this variability was also reflected in the lack of uniformity across agencies in terms of intervention models and assessment protocols. Different agencies used different procedures, and this hindered the development of a shared protocol and impeded the collection of data for research (e.g., the European Network of Investigation of Gender Incongruence [81] established partnerships with only a few Italian centers). Due to this difficulty in participating in international research programs, the Italian centers were at increased risk of isolation.

Although the agencies aimed at aligning their practices with international guidelines, many centers lacked sufficient resources, including multidisciplinary staff. In contrast, at many international centers (e.g., GIDS in London and the Center of Expertise on Gender Dysphoria in Amsterdam), psychologists work side by side with social workers, psychiatrists, endocrinologists, and other professionals. This multidisciplinary staff, in line with what international guidelines [3] recommend, guarantee for their referrals a holistic approach to care, which helps youths and their families to be well sustained and informed on all the different aspects of gender transitions. The lack of multidisciplinary staff in Italian services may indeed result in an inaccurate take in care: many respondents highlighted that, due to a lack of relevant staff, they were forced to refer families to external professionals, who often lacked knowledge and competency relating to gender incongruence. This was emphasized as a critical shortcoming, since many centers considered the presence of a severe psychiatric condition an exclusion criterion for admission. Moreover, it was frequently reported that relevant medical units were located far away, and thus families had to cope with additional burdens on their money and time to make the necessary visits for their child to be assessed. This, in turn, hindered the agencies from establishing long-term, continuous relationships with many patients, the kind of relationships that the standards of care [3,4,5] recommend for trans * youth.

Many respondents described their service as isolated, unknown to other professionals, and not recognized by society at large. They noted that, due to a lack of knowledge, parents’ first port of call was usually a private professional, who often lacked adequate knowledge of gender issues and, in line with what we have found in literature [39,40,41] some of them have been stigmatizing and transphobic. This highlights the need to promote better networks with more funding, in order to connect with services and families throughout the Italian territory. For instance, in England, the GIDS has developed a “network model of interventions” [82], whereby GIDS clinicians organize local network meetings for particularly complex referrals, which occur at different stages of treatment. Meetings are organized where the family resides and may involve other professionals involved in the care of the young person (e.g., psychiatrists, psychotherapists, psychologists from the Child and Adolescent Mental Health Services, social workers, teachers, special educational needs coordinators, heads of school, etc.). The main aim of these network meetings is to coordinate the management of the young person’s support with other local services, to offer guidance on gender issues, to facilitate communication between professionals, and to make families and professionals more aware of the young person’s needs. It would be useful to implement a similar intervention in the Italian territory, in order to better reach families who may find it difficult to maintain a long-term and continuous relationship with their nearest service. However, such an intervention would require more funding, and this is hindered by the fact that funding for the SSN is regional (whereas the London-based GIDS receives national funding) [50,51].

Notwithstanding these difficulties, the present study identified some elements of consistency among the facilities. First, all respondents endorsed an open and affirmative protocol, in line with the “watch and wait” approach of the Dutch model [72]. In general, respondents stressed the importance of “buying time” for transition decisions, suggesting a slight skepticism towards early social transitions—in contrast to the approach of some other international centers (e.g., the Child and Adolescent Gender Center Clinic at Benioff Children’s Hospital, San Francisco [83]). They emphasized that young persons and their families require space to process the changes, without direct or active intervention in support of a transition. For many respondents, ongoing psychological support for associated psychopathological conditions and family fears was deemed necessary. With respect to this latter point, the results show that parents had many fears, including a fear of transphobia, stigma, prejudice, and difficulties accepting their child’s gender incongruence; however, in recent years, these fears had begun to decrease in severity. Regarding their young patients, in line with many studies on associated conditions to gender incongruence [6,10,17,18,19,22] respondents underlined many significant psychological issues and risk factors, including isolation, eating disorders, and suicidal risk; unfortunately, not all centers could afford to provide specialized support for these conditions.

Of interest, although the presence of a severe psychological condition was usually an exclusion criterion for admission to the specialized centers, most respondents agreed on the importance of decreasing distress related to gender incongruence by promoting acceptance and fighting stigma and discrimination from both families and the broader society. This is an interesting feature of Italian services: on the one hand, respondents acknowledged that after having accessed to such services trans* youths’ mental health had improved; on the other hand, they referred that if during access an individual showed symptoms deemed too severe, he or she could not be admitted for care. Since there are no guidelines about what threshold of symptoms severity should be adopted when considering if a patient is eligible for care, we believe that this may be a problematic aspect that needs further investigation.

It is important to underline that, despite their limited resources, many agencies succeeded in building efficient networks with schools. In general, extended work with families and schools was endorsed by all respondents; this is a very positive finding that should be underlined. Families were presented as becoming more open and accepting, especially if compared to previous Italian studies (e.g., [58]), and this trend may be predominantly due to the work of the agencies, themselves, together with the increasing attention given to trans * youths by social and traditional media.

Finally, centers that were well-integrated into the SSN and thereby well-connected with other medical units benefited from an increased network of knowledge among local professionals (i.e., pediatricians, GPs, etc.). This promoted more knowledge among families and served to counteract stigma in the broader society.

## 5. Conclusions

Our findings highlighted a lack of an effective multidisciplinary care in many services and some shortcomings in the network between services. As suggested by international guidelines [3,4,5], multidisciplinarity is a key aspect for the care of trans * youth, and thus should be implemented in Italian specialized services as well. Specialized training should be promoted to form prepared medical and psychological staff (endocrinologists, psychologists, psychiatrists, voice specialists, etc.) to work side by side in the centers. Our hope is that future efforts will build a more efficient network of services with stable connections to the SSN, external medical units, and both national and international research centers.

Furthermore, the peculiarity of Italian territory (e.g., discrepancies between regions) leads to the need of implementing a network model of interventions to reach families in every area and fighting isolation and stigma suffered by the youths. Moreover, sensitization projects should be implemented to provide knowledge and information to primary care providers and medical professionals (e.g., pediatricians) which often resulted to be the first contact for families.

Finally, even if there is a need for congruence among the specialized centers concerning the support policies and interventions provided for gender diverse children and adolescents, at the same time, with regards to gender incongruence, it is highly important to consider that each person is singular, and each referral has a complex, unique, and authentic psychological background. Each intervention, in the framework of a multidisciplinary care, should be exclusively tailored to the individual child or adolescent.

### Limitations of the Study

Some limitations of the present study warrant mention. First, the interviews captured only subjective points of view, and thus they may not reflect the ideas of other professionals, even in the same agency. Second, the research did not include all Italian specialized centers; thus, the study was exploratory and not representative of the national situation. Thirdly, the interviews were conducted over a two-year timespan (2017–2019); during those years, many changes occurred in the services, thus some shortcomings found initially may have been overcome. Finally, although the qualitative research generated deep insights into the work, assessment protocols, and intervention models of these facilities, as already outlined, the insights were determined from only a relatively small sample.

## Figures and Tables

**Table 1:** Interview themes and subthemes.

Theme	Subtheme
1. Source of referrals	a. Prevalence of Internet or word-of-mouth referrals b. Lack of consistency between services c. Recent increase in AFABs
2. Assessment protocol and intervention model	a. Use of a “watch and wait” model b. Adherence vs. non-adherence to the guidelines
3. Psychological support (for youths and families)	a. Work with families b. Work on associated psychopathology c. Work with other services (e.g., schools)
4. Agency shortcomings	a. Specific to region or territory b. Lack of network between services c. User requests to speed up referrals

**Table 2:** Transcript codes for professionals.

Professional Working in…	Code
Specialized center in the north of Italy	SC, N
Specialized center in the center of Italy	SC, C
Specialized center in the south of Italy	SC, S
Association in the north of Italy	A, N
Association in the center of Italy	A, C
